# The Therapeutical Applications of Peroxide of Hydrogen, Glycozone, Hydrozone, and Eye Balsam

**Published:** 1896-09

**Authors:** 


### The Therapeutical Applications of Peroxide of Hydrogen (Medicinal) Glycozone, Hydrozone, and Eye Balsam. By Charles Marchand, Chemist Graduate of the Ecole Central des Arts et Manufactures de Paris (France). Treatment of Diseases Caused by Germs, Bacteria, Microbes. Eleventh edition, 215 pages, 28 Prince Street, New York, 1896.

This book has been noticed in these pages before. As its name indicates it gives the results of treatment with Peroxide of Hydrogen.

It opens with a reference to the orgin of bacteriology in the discoveries of Professor Pasteur with regard to the silk-worm disease.

Then follows a list of diseases, due to germs or microbes, arranged under a suitable system of classification. The destructive action of ozone upon these organizations is then dwelt upon in the next two pages. Says the author, in speaking of ozone, "a fact known by bacteriologists and chemists is that all virus is albuminoid, whether propagative or not, it is destroyed, or by coagulation rendered inert by the oxydizing action of ozone."

Then follows a table, highly interesting to chemists of the comparative chemical reactions between hydrozone and ozone, showing conclusively that hydrozone fulfills all the functions of ozone.

A description of hydrozone follows and a detailed description of the action of hydrozone upon open surfaces in a diseased state.

A chapter on glycozone and its nature and effects is given, with a special chapter on its therapeutic uses by Dr. Cyrus Edson of New York.

The use of these various preparations in ozena, hay fever, la grippe, asthma, bronchitis, laryngitis, pharyngitis, croup, whooping cough, consumption, the various throat diseases, especially diphtheria and scarlatina-anginosa.

A series of diagrams shows the method of using a spray of these preparations in throat troubles and then follows the treatise on the internal use of these several preparations.

The treatment of typhoid fever is by Dr. Elmer Lee, of Chicago, and that of cholera and yellow fever by Dr. Edson.

The use of "eye balsam" on the eyes is adverted to, along with its value for the ear. This "balsam" is described and then comes a chapter upon its use in dental surgery.

The author claims a field of usefulness for it in cancerous sores.

Much more than is here noted is contained in the book, but it is much too voluminous for further comment. The best way is to procure the book and read it. This may be readily done, for the author says he will send a copy free to every reader of THE HOMŒOPATHIC PHYSICIAN, who will write for it, and mention this journal.

